# EUS-guided ethanol ablation of metastatic functional insulinoma

**DOI:** 10.1016/j.vgie.2021.05.013

**Published:** 2021-06-24

**Authors:** Ahmed Altonbary, Hazem Hakim, Wagdi Elkashef

**Affiliations:** 1 Department of Gastroenterology and Hepatology, Mansoura Specialized Medical Hospital, Mansoura University, Mansoura, Egypt; 2 Department of Pathology, Mansoura University, Mansoura, Egypt

**Keywords:** EA, ethanol ablation, NET, neuroendocrine tumor

## Abstract

Video 1 EUS-guided ethanol ablation of metastatic functional insulinoma.

EUS-guided ethanol ablation of metastatic functional insulinoma.

## Introduction

Pancreatic neuroendocrine tumors (NETs) are rare, representing only 1% of all pancreatic tumors.^1^ Pancreatic NETs are classified as functional or nonfunctional depending on the presence or absence of a clinical, hormonal hypersecretion syndrome. The clinical management of these lesions is challenging.

For functioning pancreatic NETs, the goal of treatment is to induce necrosis of most of the tumor cells to mitigate hormonal hypersecretion, with cessation of symptoms.^2^ Over the past 2 decades, EUS-guided intervention has evolved from a diagnostic method to a therapeutic modality. Because EUS-guided treatment is effective and safe, it can be an alternative for patients with pancreatic NETs, especially for those who are poor surgical candidates or refuse surgery.^3^ Herein, we describe a case of EUS-guided ethanol ablation (EA) of metastatic functional insulinoma.

## Case

A 44-year-old man with recurrent attacks of hypoglycemia requiring intravenous glucose infusion was referred to our facility for EUS evaluation of suspected insulinoma. His laboratory values showed elevated serum insulin (35 mIU/L) and C-peptide levels with low serum glucose (40 mg/dL) during the hypoglycemic attacks. However, abdominal CT performed in another facility could not localize the suspected NET.

**Figure 1:**
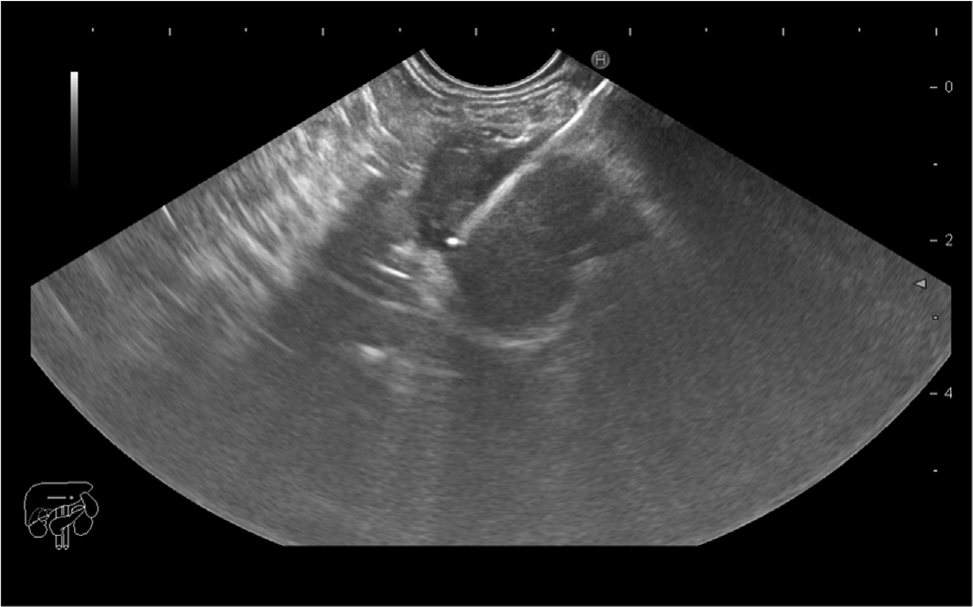
EUS-guided fine-needle biopsy of the pancreatic neck mass.

EUS examination using a Pentax linear Echoendoscope EG3870UTK (Pentax Medical, Tokyo, Japan) attached to a Hitachi Avius ultrasound system (Hitachi Medical Systems, Japan) revealed a hypoechoic pancreatic neck mass measuring about 19 × 18 mm without any vascular invasion. On routine liver scan during examination, a small (5-mm) hypoechoic lesion was noticed in the left liver lobe. EUS-guided fine-needle biopsy was performed using a 22-gauge needle (Acquire needle, Boston Scientific, Natick, Mass) and the slow pull technique (Fig. 1); both pancreatic and hepatic lesions underwent biopsy using the same needle.

**Figure 2:**
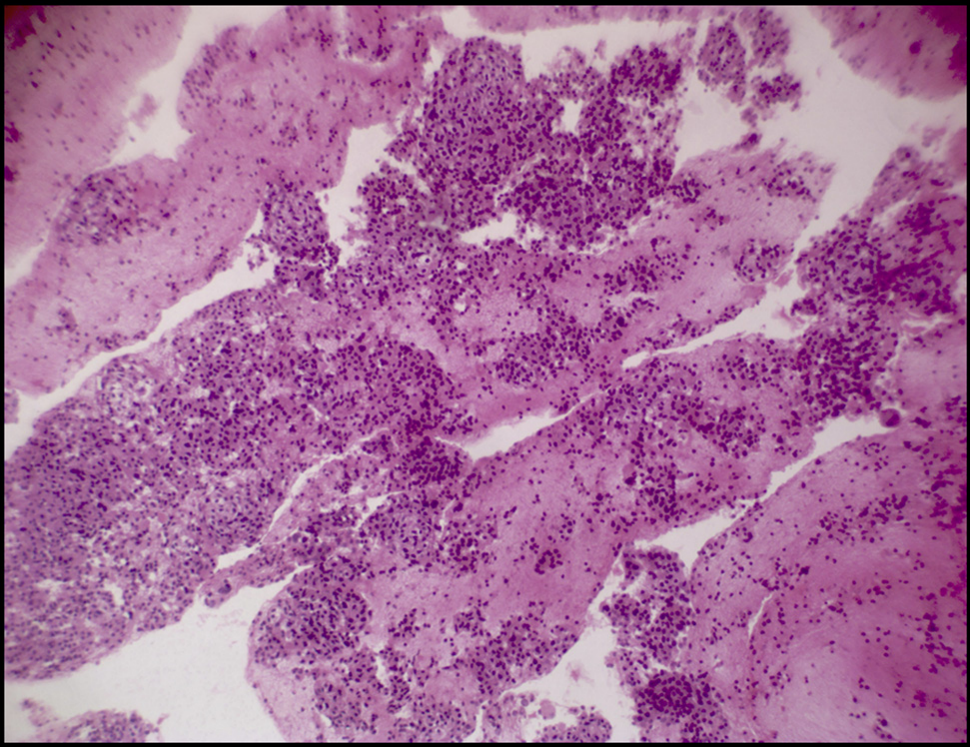
Small tumor cells with central nuclei (hematoxylin and eosin, orig. mag. ×100).

**Figure 3:**
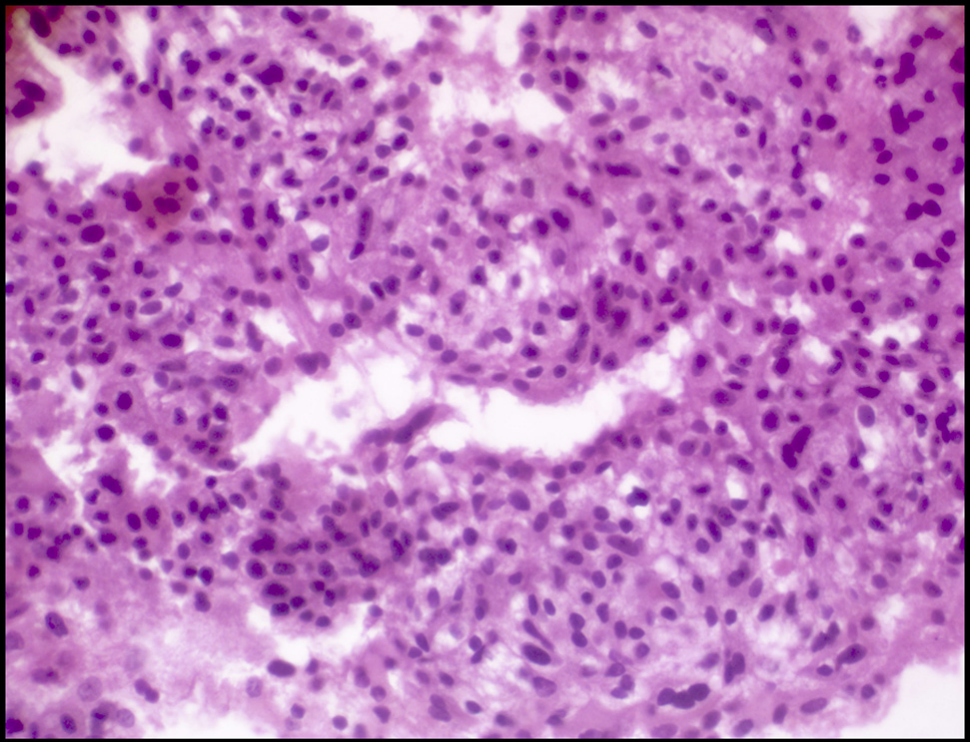
Small tumor cells with mild pleomorphism (hematoxylin and eosin, orig. mag. ×100).

**Figure 4:**
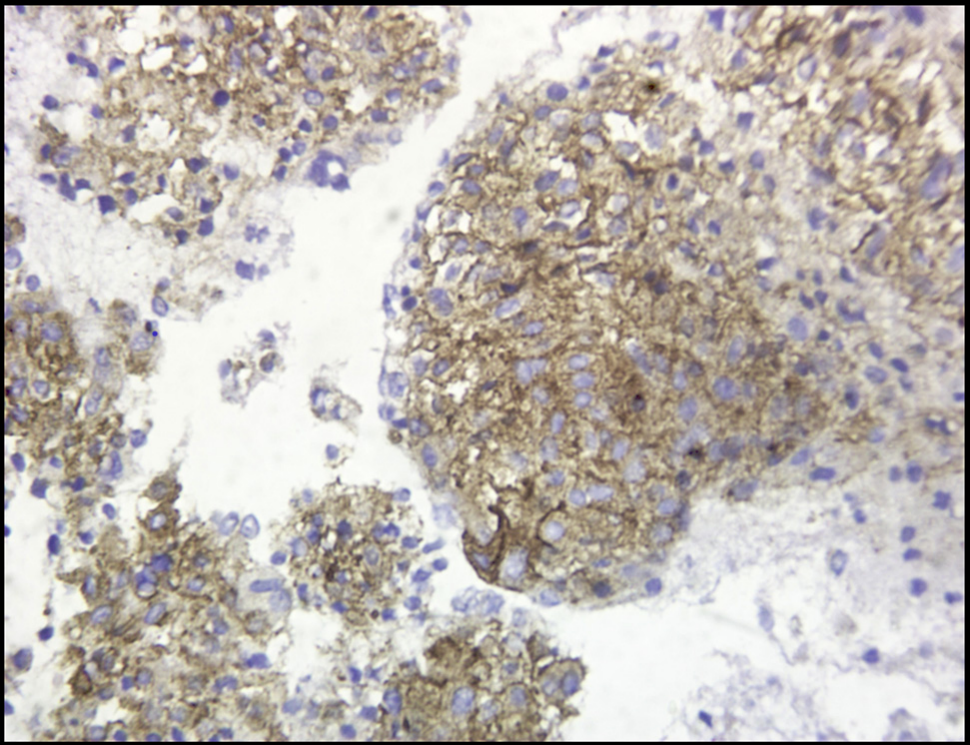
Positive cytoplasmic reaction to synaptophysin.

**Figure 5:**
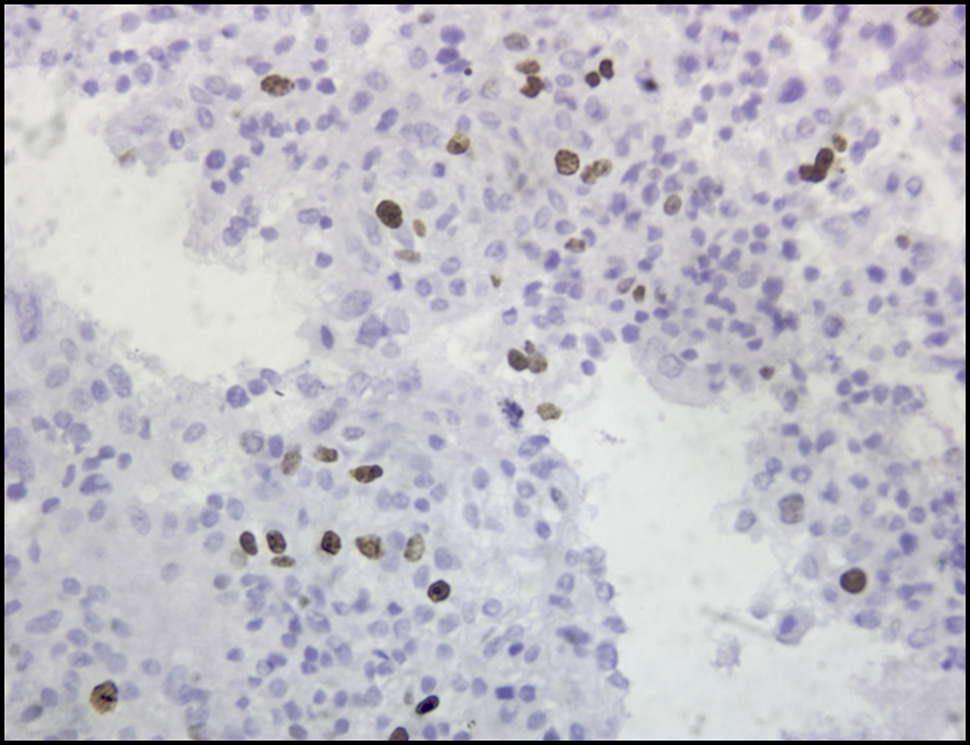
Positive nuclear reaction to reaction to Ki-67 with low proliferation index (about 5%).

Cytopathologic examination revealed small tumor cells with central nuclei (hematoxylin and eosin, original magnification ×100) (Fig. 2) and small tumor cells with mild pleomorphism (hematoxylin and eosin, original magnification ×400) (Fig. 3) from both pancreatic and hepatic lesions; positive cytoplasmic reaction to synaptophysin (Fig. 4); and positive nuclear reaction to Ki-67 with a low proliferation index (about 5%) (Fig. 5). Findings were consistent with metastatic insulinoma. The patient refused any surgical intervention, and the decision was made to proceed with EUS-guided EA for the primary pancreatic lesion. It was believed that the small hepatic lesion would also be considered for ablation if hypoglycemic symptoms persisted.

## Set-up

Our set-up is as follows:

• Ethanol 95% to 99% is most preferred.
• Both 22-gauge and 25-gauge EUS needles can be used.
• A 1.0 to 3.0 mL syringe is used for precise injection.
• Mixing lipiodol can be helpful in evaluating complete response on CT after the procedure.
• Antibiotics are not recommended before or after the procedure.

**Figure 6:**
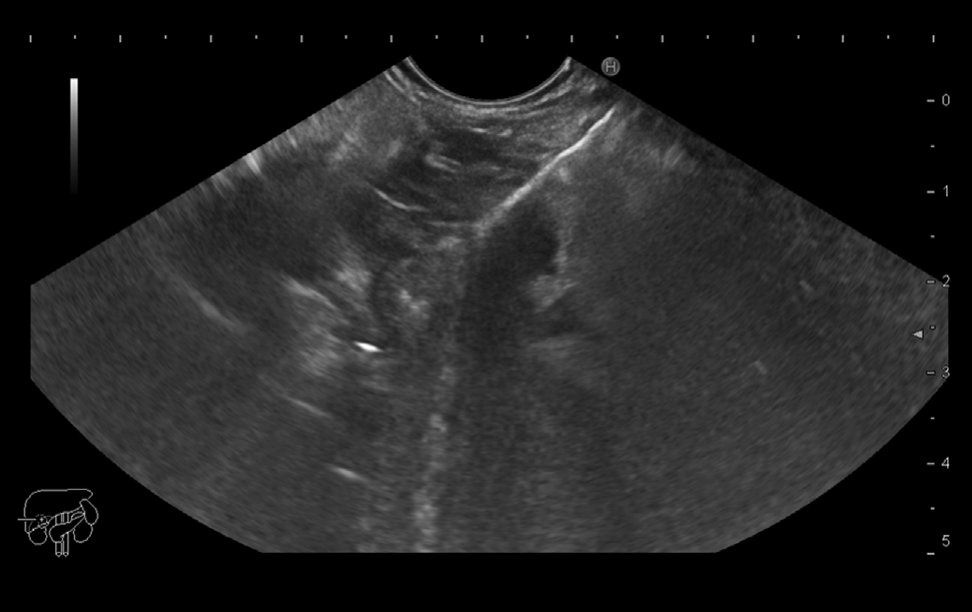
Ethanol injection with observation of hyperechoic blush under EUS in real time.

After Doppler examination, the center of the lesion was punctured with a 22-gauge needle (Expect needle, Boston Scientific) for precise control of injection. For ablation, 2 mL of 95% ethanol was determined to be the maximum volume according to the following formula: (Major axis + Minor axis of the tumor)/2.^4^ While the needle was being withdrawn, 1 mL was slowly injected, with observation of hyperechoic blush under EUS guidance in real time extending proximally to the periphery of the tumor. Injection was terminated abruptly to avoid leakage beyond the tumor borders (Fig. 6).

Based on the pattern of spread after initial injection, an additional injection of 1 mL was performed in the upper part of the lesion with the same technique until the hyperechoic blush extended to most of the lesion (Video 1, available online at www.giejournal.org).

The patient was discharged after being monitored for 4 hours and resumed oral feeding the same night with monitoring of blood glucose level at home. No adverse events occurred after the ablation, including abdominal pain, fever, or pancreatitis, and no episodes of hypoglycemia were reported for 6 months after ablation.

Key technical points:

• Keep the needle inside the tumor during injection.
• Observe hyperechoic blush under EUS guidance in real time.
• Do not inject too much ethanol (2 mL maximum in each session).
• Minimize ethanol leakage into the surrounding normal area.

## Discussion

EUS-guided interventions for functioning NETs include EUS-guided radiofrequency ablation and EUS-guided EA. Published data from 2006 on EUS-guided EA of functioning insulinomas included 18 patients. All cases showed symptomatic improvement after EA, and mild pancreatitis was the most reported adverse event.^3^

In conclusion, EUS-guided EA of a functioning insulinoma is feasible, minimally invasive, effective, and safe, with an acceptable level of postprocedural adverse events. This technique might be applied to a wider range of potential candidates with poor general condition or those refusing surgical treatment. EUS can also detect small hepatic metastases missed by CT, with feasible EUS-guided fine-needle biopsy even in small lesions of <5 mm.

## Disclosure

*All authors disclosed no financial relationships.*

## Supplementary data

Video 1 EUS-guided ethanol ablation of metastatic functional insulinoma.
